# Elevated Temperatures Impose Transcriptional Constraints and Elicit Intraspecific Differences Between Coffee Genotypes

**DOI:** 10.3389/fpls.2020.01113

**Published:** 2020-07-21

**Authors:** Raphael Ricon de Oliveira, Thales Henrique Cherubino Ribeiro, Carlos Henrique Cardon, Lauren Fedenia, Vinicius Andrade Maia, Barbara Castanheira Ferrara Barbosa, Cecílio Frois Caldeira, Patricia E. Klein, Antonio Chalfun-Junior

**Affiliations:** ^1^Plant Physiology Sector, Biology Department, Universidade Federal de Lavras (UFLA), Lavras, Brazil; ^2^Department of Horticultural Sciences, Texas A&M University, College Station, TX, United States; ^3^Forest Sciences Department, Universidade Federal de Lavras (UFLA), Lavras, Brazil; ^4^ Institute for Plant Genomics and Biotechnology, Texas A&M University, College Station, TX, United States

**Keywords:** coffee breeding, global warming, energetic homeostasis, intraspecific variation, RNAseq analysis, sugar metabolism

## Abstract

The projected impact of global warming on coffee production may require the heat-adapted genotypes in the next decades. To identify cellular strategies in response to warmer temperatures, we compared the effect of elevated temperature on two commercial *Coffea arabica* L. genotypes exploring leaf physiology, transcriptome, and carbohydrate/protein composition. Growth temperatures were 23/19°C (day/night), as optimal condition (OpT), and 30/26°C (day/night) as a possible warmer scenario (WaT). The cv. Acauã showed lower levels of leaf temperature (Tleaf) under both conditions compared to cv. Catuaí, whereas slightly or no differences for other leaf physiological parameters. Therefore, to explore temperature responsive pathways the leaf transcriptome was examined using RNAseq. Genotypes showed a marked number of differentially-expressed genes (DEGs) under OpT, however DEGs strongly decrease in both at WaT condition indicating a transcriptional constraint. DEGs responsive to WaT revealed shared and genotype-specific genes mostly related to carbohydrate metabolism. Under OpT, leaf starch content was greater in cv. Acauã and, as WaT temperature was imposed, the leaf soluble sugar did not change in contrast to cv. Catuaí, although the levels of leaf starch, sucrose, and leaf protein decreased in both genotypes. These findings revealed intraspecific differences in the underlying transcriptional and metabolic interconnected pathways responsive to warmer temperatures, which is potentially linked to thermotolerance, and thus may be useful as biomarkers in breeding for a changing climate.

## Introduction

Climate change is multifaceted and despite measurable impacts of elevated temperatures on agriculture (Lobell et al., 2011; Zhao et al., 2017), there remains considerable gaps on how coffee systems will be affected by both short- and long-term changes in the environment. Several studies on the impact of climate change on coffee systems have projected marked negative effects on yield, berry quality, suitable planting areas, and incidence of disease and insects (reviewed by DaMatta et al., 2019). Collectively, these environmental stresses will likely impose both economic and social problems within many coffee producing regions (Bunn et al., 2014; Bunn et al., 2015). Despite attenuating factors associated with increasing global CO_2_ levels that could partially mitigate the negative production trends described above (Rodrigues et al., 2016) and numerous studies demonstrating the impact of temperature on coffee physiology (Drinnan and Menzel, 1995; DaMatta and Ramalho, 2006; Läderach et al., 2017), a detailed understanding of the molecular mechanisms in response to warmer temperature is lacking.

*C. arabica* L. is a tropical tree responsible for the major worldwide production of coffee (ICO, 2019) and its optimal growth temperature is considered between 18 and 23°C (Camargo, 1985; Teketay, 1999). The coffee tree has a periodicity growth habit that closely follows rainfall patterns and, historically, it is considered highly sensitive to climatic changes, especially temperature and drought (DaMatta and Ramalho, 2006; Camargo, 2010; DaMatta, 2018). Mean temperatures are projected to increase by 2.6–4.8°C (IPCC, 2013; IPCC, 2014), which may have serious repercussions on coffee production. Considering these changing temperatures, select genotypes were identified that outperformed others when exposed to higher annual mean temperatures (Damatta et al., 2018; Marie et al., 2020). This suggests there is potentially useful intraspecific variability of thermotolerance in some genotypes and investigation into the molecular mechanisms underlying this variability is warranted (DaMatta, 2018).

Increasing temperature impacts plant physiology from the cellular to the whole plant level and changes photoassimilate allocation to repair and recovery processes (Bita and Gerats, 2013; Bokszczanin et al., 2013; Marias et al., 2017a). However, the stress severity depends on intensity and duration of exposure beyond the plant species and within genotypes (Teskey et al., 2015). Therefore, beyond particular characteristics and growth conditions, a comprehensive effect of increasing temperature on plants needs first to differentiate data from a moderate long-term change to more drastic ones such as short-duration heat-waves (Thornton et al., 2014). Both phenomena are predicted to be more frequent in the future and may occur singly or concomitantly (Hao et al., 2013; IPCC, 2014) highlighting the need for independent and overlapping studies.

In plants, thermoregulation is the ability to alter thermogenic properties and maintain constant temperature under fluctuating environmental temperatures, an important feature related to development, in special reproductive organs, and attraction of pollinators (Minorsky, 2003; Watling et al., 2008). Recently, advances have updated this topic being proposed that leaf and photosynthetic traits evolved to promote a limited homeothermy and maximize instantaneous and lifetime carbon gain in space and time across variable temperature regimes (Michaletz et al., 2015; Michaletz et al., 2016). Thus, thermoregulation is a very important aspect to be considered in environmental interactions studies, especially for crops due to its impact on growth rates, vegetation dynamics, and reproductive development (Jagadish et al., 2016; Janni et al., 2020). To understand the limits of coffee thermotolerance, recent studies have explored the effect of a gradual increasing temperature or extreme heat stress on select physiological processes (reviewed by DaMatta et al., 2019). Minimal impact on photosynthetic-related parameters was observed when various coffee genotypes were exposed to temperatures up to 37°C whereas maximum photosynthetic damage occurred at 42°C for all coffee genotypes (Martins et al., 2016). Although coffee presents moderate thermotolerance of photosynthetic-related processes, most genotypes produced abnormal reproductive structures at these elevated temperatures (DaMatta et al., 2019). Accordingly, coffee plants subjected to 45°C for 1–1.5 h showed leaf age-related differences in physiological recovery and did not bear flowers or fruits (Marias et al., 2017a). These results demonstrate that, depending on the tissue and stage of plant development, coffee thermotolerance may be substantial regarding physiological parameters.

From the molecular point of view, temperature has well-documented effects on tissue rates of metabolism and physiology (reviewed by Michaletz et al., 2015) and it is perceived by multiple pathways in model plants and crops (Wigge, 2013; Hasanuzzaman et al., 2013; Ibañez et al., 2017). A general and critical cellular response to heat stress is the activation of heat shock proteins (HSPs), which function as chaperones ensuring proper folding of proteins (Ohama et al., 2016). Importantly, phytochromes act as thermosensors joining the related processes of light perception to temperature (Jung et al., 2016). However, the impact of elevated temperature on gene expression and associated thermotolerance is highly heterogeneous in plant species (von Koskull-Döring et al., 2007; Ohama et al., 2017), which in the last instance may affect plant development and cause many phenotypic variations (Atkin et al., 2006; Scheepens et al., 2018). Therefore, extrapolation of molecular mechanisms relating to thermotolerance in model species is unreliable and will require direct validation.

At present, molecular studies examining the effect of elevated temperature on *Coffea* sp. are limited when compared to molecular-based drought studies in this crop (Moffato et al., 2016; DaMatta et al., 2019). One of the few studies demonstrated that the allotetraploid *Coffea arabica* presents a higher phenotypic homeostasis compared to the diploid parents, *C. canephora* and *C. eugenioides*, in response to different temperature conditions (Bertrand et al., 2015). However, to the best of our knowledge, a large-scale analysis of the intraspecific transcriptional variation in response to elevated temperature has not been reported. Thus, contrasting *Coffee arabica* L. genotypes could reveal differences at thermotolerance molecular pathways and important strategies toward breeding programs.

Thermotolerance is acquired *via* protective cellular machinery gained throughout coffee plant maturation (Marias et al., 2017a; Marias et al., 2017b) as also demonstrated for other plant species (Wollenweber et al., 2003; Hedhly et al., 2009; Scheepens et al., 2018). This suggests that young plants are more sensitive and require long-term exposure to stress to acclimatize making this stage in plant development a useful model to examine the impact of warmer temperatures on gene expression during acclimation. Thus, to study intraspecific variation associated with mechanisms of thermotolerance on coffee, the present study imposed elevated temperatures on 1-year old plants of two coffee genotypes, cv. Catuaí IAC 144 and cv. Acauã, which have been reported to be contrasting for agronomic traits including temperature responses (Carvalho, 2008). Physiological parameters were evaluated as well as a global transcriptional analysis in conjunction with an initial metabolomics investigation of photo-assimilates, sugars, and protein.

## Material and Methods

### Plant Material

Two *Coffea arabica* genotypes, cvs. Acauã and Catuaí IAC 144, hypothesized to differ in heat tolerance (Carvalho, 2008) were examined in the present study. Coffee plants were cultivated in growth chambers with 12 h of light, 60% humidity and either 23/19°C or 30/26°C (day/night temperatures) that are considered the optimal (OpT) and warm temperatures (WaT), respectively (DaMatta and Ramalho, 2006). For the RNAseq and RT-qPCR analyses, plants were obtained from 200 seeds of each genotype cultivated for 30 d in a commercial substrate (Professional Growing Mix, Sun-Gro Horticulture). After 30 d, seedlings were individually transplanted to a two-liter-pot and maintained in greenhouses (Department of Horticultural Sciences, Texas A&M University, USA) with 50% shade until the three-leaf pair stage. At the three paired leaf stage, plants were transferred to the Texas A&M AgriLife Research and Extension Center (Overton, TX), randomized in complete blocks with split plot restrictions. Plants were allowed to acclimate under controlled growth conditions for 15 d at OpT. Acclimated coffee plants were then divided between two chambers at either OpT or WaT conditions and maintained for 4 weeks. Each biological repetition was composed of five excised leaves that were harvested immediately and placed in liquid nitrogen, pulverized with a mortar and pestle, and subsequently stored at −80 °C until analysis.

For gas exchange and sugar content analyses, the experiment was repeated using similar age plants of each genotype transferred from greenhouses to a Conviron^®^ growth chamber (Plant Physiology Sector, Federal University of Lavras, Brazil). Twenty plants were transplanted to a mix of soil, sand, and fertilizer formula 5–25–15 of N-P-K and maintained in a greenhouse for 1 week. Then, they were transferred to a chamber, acclimatized for 2 weeks at OpT under a 12 h light/12 h dark photoperiod. Subsequently, plants were maintained at OpT or at WaT for 4 weeks.

### Gas Exchange Measurements

Physiological parameters, such as carbon assimilation rate, stomatal conductance, transpiration rate, and chlorophyll fluorescence were measured for each coffee genotype at different temperatures and maintained between the second and fourth hours of the light period on completely expanded leaves. Ten plants of each cultivar were randomly selected, and one leaf from each used for measurements with a portable infrared gas analyzer IRGA (LI-6400XT, LI-COR^®^) once a week for 4 weeks. These measurements were done with reference CO2 concentration fixed at 400 µM mol^−1^, relative humidity was set to 60% and photon flux density inside the measuring chamber to 1,000 µmol m^−2^ s^−1^.

### RNAseq Library Preparation

Five biological repetitions for each genotype at the two growth temperature regimes were used (20 RNAseq libraries). The RNA extractions were performed with 100 mg of powdered tissue using the ConcertTM Kit Plant RNA Reagent (Invitrogen^®^) and followed by treatment with the Turbo DNA-free Kit (Ambion^®^). RNA integrity and purity were assessed by 1% agarose gel electrophoresis and analyzed on a DeNovix DS-11 spectrophotometer (DeNovix Inc., Wilmington, DE, USA) and a Bioanalyzer 2100 (Agilent Technologies, Santa Clara, CA). All samples presented standard values and RNA integrity number (RIN) higher than 7.0. The TruSeq library preparations were constructed using the cDNA Synthesis kit (Illumina Inc., San Diego, CA, USA). Two lanes of paired-end (2x150 bp) sequencing of the cDNA libraries were performed on the Illumina HiSeq 2000 (Illumina Inc., San Diego, CA, USA). Library preparation and sequencing were performed by AgriLife Genomics and Bioinformatics Services (Texas A&M University, College Station, TX, USA) in April 2015. Sequence cluster identification, quality prefiltering, base calling and uncertainty assessment were done in real-time using Illumina’s HCS 2.2.58 and RTA 1.18.64 software with default parameter settings. All the reactions followed the respective manufacturer’s instructions. Pre-processed libraries are available in SRA under BioProject ID PRJNA609253.

### RNAseq Analysis

Approximately 183 million sequenced paired-end reads were used for alignment against the *Coffea canephora* genome (available at http://coffee-genome.org) using the STAR v. 2.5.3a aligner with default parameters. Libraries were sorted and PCR duplicates were removed with Picard tools. Approximately 115 million paired-end reads were uniquely mapped to exons and read counts were quantified with htseq-count script. For differential expression analyses the library WAT_AC_1 (cv. Acauã at WaT conditions, replicate 1) was not considered due to a relative low number of uniquely mapped reads (~2.3 million). Differentially expressed genes (DEGs) of the same cultivar in contrasting temperature conditions were identified using the Bioconductor R package edgeR by comparing the normalized number of reads aligned to each gene model in different conditions using a Generalized Linear Model applied to the expression matrix (Robinson et al., 2010; Huber et al., 2015). Benjamini and Hochberg’s false discovery rate (FDR) below 0.05 and a minimum log2 fold change of one were the parameters used to consider a gene differentially expressed between the two conditions. To improve the quality of functional characterization of the DEGs, their respective protein sequences were subjected to homology searches with BLASTP version 2.7.1+ (Camacho et al., 2009) against all plant proteins in the National Center of Biotechnology Information (NCBI) non-redundant protein database (nr). In addition, we enriched our DEG results by mapping those proteins against the Kyoto Encyclopedia of Genes and Genomes (KEGG) database with the BlastKOALA tool (Kanehisa et al., 2016) in order to find the pathways that the DEGs were related to.

### Quantitative Gene Expression Analysis (RT-qPCR)

RT-qPCR analysis was conducted from three biological repetitions with two technical replicates for each genotype at both growth temperatures. Total RNA was isolated using 100 mg of frozen powdered tissue and the PureLink™ Plant RNA Reagent System (Thermo Fisher^®^, Invitrogen) according to the manufacturer’s protocol. Samples were subsequently treated with the Turbo DNA-free Kit (Ambion^®^) for removal of DNA contamination. RNA purity was analyzed on a DeNovix DS-11 spectrophotometer (DeNovix Inc.). First-strand cDNA was synthesized using SuperScript^®^ III First-Strand Synthesis System (Invitrogen™) according to the manufacturer’s protocol. RT-qPCR reactions were conducted using SYBR Green MasterMix (Applied Biosystems^®^) following the manufacturer’s instructions. Gene-specific primers (**Table S2**) were designed in non-conserved regions using the Primer-BLAST tool (Ye et al., 2012) with primer specificity validated using the CoffeeHub and Phytozome databases (Goodstein et al., 2012; Denoeud et al., 2014). Primer efficiency and RT-qPCR analyses were performed using the CFX384 Touch™ Real-Time PCR Detection System (Bio-Rad Laboratories, Hercules, CA, USA). Differential gene expression analysis was inferred using an adapted modeling approach under delta Cycle Threshold (dCt) values (Yuan et al., 2006) in relation to the reference genes *Malate dehydrogenase* (*MDH*, GW464198.1) and *Ubiquitin-conjugating enzyme E2* (*UBQ2*; GR984245) previously described and validated for RT-qPCR in *Coffea* spp. (Martins et al., 2017).

### Carbohydrate and Protein Content

Carbohydrate and protein content analyses were conducted from four biological repetitions with two technical replicates for each genotype at the two growth temperatures. The extraction of carbohydrates and proteins was based on Zanandrea et al. (2010) with modifications (Silva et al., 2014) in which 1,000 mg of frozen powdered tissue (fresh weight) were homogenized in 5 ml of 100 mM potassium phosphate buffer (pH 7.0) and then placed in a water bath for 30 min at 40°C. The solution was centrifuged at 10,000 g for 10 min and the supernatant was collected. The process was repeated twice, and supernatants were combined totalizing 10 ml. For extraction of starch, the pellet was resuspended in 10 ml of 200 mM potassium acetate buffer (pH 4.8) and 16 units of amyloglucosidase enzyme were added. Then, samples were incubated in a water bath at 40°C for 2 h. Following centrifugation at 10,000 g for 20 min, the supernatant was collected for measurements. Starch, sucrose, and total soluble sugars were quantified as described by Dische (1962), and the level of reducing sugars was quantified according to Miller (1959). Protein was quantified as described by Bradford (1976) and analyzed in a spectrophotometer at 570 nm comparing results with a standard curve of 0.1 μmol/ml Bovine Serum Albumin (BSA).

### Statistical Analysis of Physiologic, Metabolic, and RT-qPCR Expression Data

The modeling approach was carried out by linear mixed models (LMM) using the “lmer” function from the lme4 R package (Bates et al., 2015) for the statistical analysis of IRGA physiological data, metabolic parameters, and RT-qPCR expression. In all experiments, the individuals were used as random factors to deal with the dependence between observations at the same individual across different weeks or conditions. Additionally, the models were fitted by maximum likelihood. The treatments were coded as a factor level and used as fixed effects including temperature conditions (WaT or OpT), cultivar (Catuaí or Acauã), and weeks (only for the physiological analyses), in cases of interest, the interactions between the fixed effects were accounted. Residuals normality and variance homogeneity were assessed by Shapiro-Wilk test and residuals versus fitted plots, respectively. The *post hoc* pairwise contrasts between factor levels were obtained by “lsmeans” function from “lsmeans” package (Lenth, 2016) using Tukey adjust method. Statistical significance was assessed using Satterthwaite approximation, by the package lmerTest (Kuznetsova et al., 2015). For the RT-qPCR analysis, genes were considered differentially expressed if their expression profile respected three parameters: 1) the residuals of modeled Cycle Threshold (Ct) values presented a normal distribution, 2) pairwise differences of two contrast conditions (i.e. WaT Acauã plants against OpT Acauã plants) presented a Tukey adjusted p-value<0.05, and 3) the expression mean of a given gene was at least 2 times more expressed in one of the compared conditions (−1 < log2FC > 1).

## Results

### Coffee Genotypes Present Physiological Differences in Response to Warm Temperatures

**Figure 1:**
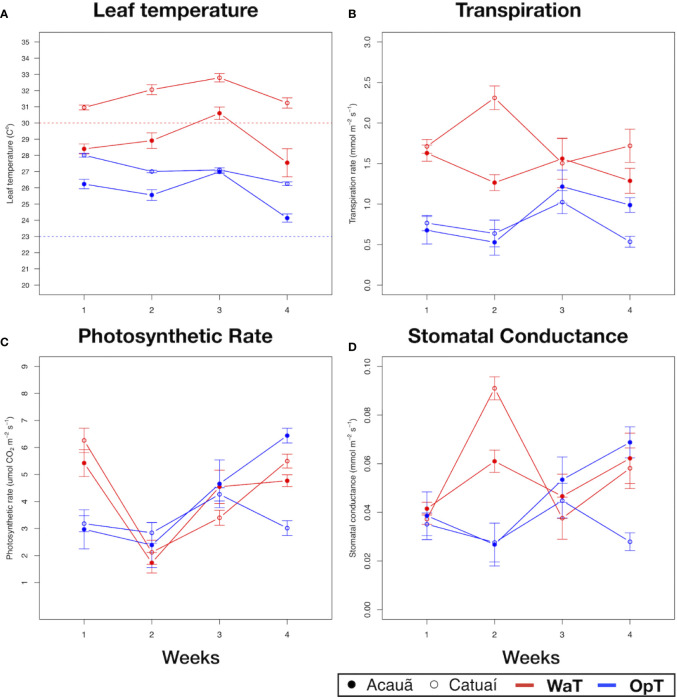
Physiological analysis of two coffee genotypes under optimal and warm temperatures. Physiological parameters of two coffee genotypes, cv. Acauã (closed circles) and cv. Catuaí (open circles), were measured along 4 weeks for each temperature condition, 23/19°C (OpT) and 30/26°C (WaT). **(A)** Leaf temperature (Tleaf). Dotted lines in the figure represent the chamber ambient temperature, OpT (blue dotted line) or WaT (red dotted line), at the time measures were made; **(B)** transpiration rate; **(C)** photosynthetic rate; **(D)** stomatal conductance. Labels: OpT, optimal temperature (23/19°C, day/night); WaT, warm temperature (30/26°C, day/night). Each point represents the mean of 10 plants. Error bars depicts the standard error.

Physiological analyses showed that coffee genotypes, cv. Catuaí and cv. Acauã, present similar trends under OpT and also in response to WaT, however quantitative and transient differences were observed for the two genotypes during the 4-week experiment (**Figure 1** and **Table S1** for statistical analyses). The main quantitative difference was noted for Tleaf where cv. Catuaí consistently had higher temperature values than cv. Acauã independent of the imposed conditions (**Figure 1A**).

At OpT conditions, both genotypes had Tleaf above 23°C throughout the 4-week treatment, however, cv. Catuaí kept Tleaf between 26–28°C whereas Acauã always had a lower Tleaf than cv. Catuaí, except at week 3 under OpT (**Figure 1A** and **Table S1**). These differences in Tleaf were not correlated with leaf transpiration as both genotypes showed similar values at OpT (**Figure 1B**). From this, we concluded that plants of cv. Catuaí, in general, presented a basal temperature higher than cv. Acauã. As plants were subjected to WaT conditions, Tleaf gradually increased for both coffee cultivars during the first 3 weeks of elevated temperatures and then decreased for both cultivars (**Figure 1A**). As was observed under OpT conditions, cv. Catuaí showed higher Tleaf values compared to cv. Acauã. Catuaí always remained above the 30°C imposed by chamber ambient temperature whereas cv. Acauã showed Tleaf mostly below 30°C.

In examining leaf transpiration at WaT, both cultivars displayed an increase in transpiration over that observed at OpT (**Figure 1B**). A transient difference in transpiration was observed at week 2 with cv. Catuaí showing a marked increase, but this difference was not significant and did not persist into the subsequent weeks under elevated temperature (**Table S1**). Regardless, Tleaf and transpiration values did not correlate well since, in general, lower Tleafs are associated with evaporative cooling driven by higher transpiration rates. This poor correlation is especially apparent when examining the results observed at week 2 under WaT conditions; transpiration rate and Tleaf for cv. Catuaí was markedly higher compared to cv. Acauã (**Figures 1A, B** and **Table S1**). We propose that cv. Catuaí juvenile trees have a lower efficiency to control temperature because Tleaf was higher than cv. Acauã under both OpT and WaT growth conditions.

Additionally, photosynthetic rates and stomatal conductance did not show consistent differences across time points between the coffee genotypes (**Figures 1C, D** and **Table S1**). For instance, comparing the two genotypes in each condition a similar trend can be noted, except at week 4 for OpT conditions where a significant difference was observed (**Figure 1C** and **Table S1**). For stomatal conductance, consistent differences between the two genotypes were not apparent since stomatal conductance varied only at week 4 in OpT (**Figure 1D** and **Table S1**). These results suggest that, despite apparent differences in their control of temperature, the coffee genotypes examined did not show consistent differences in photosynthesis or stomatal conductance during the 4 weeks of elevated growth temperature.

### Transcriptional Pathways Related to Energy Metabolism Are Affected by Warmer Temperatures in a Genotype-Dependent Manner

**Figure 2:**
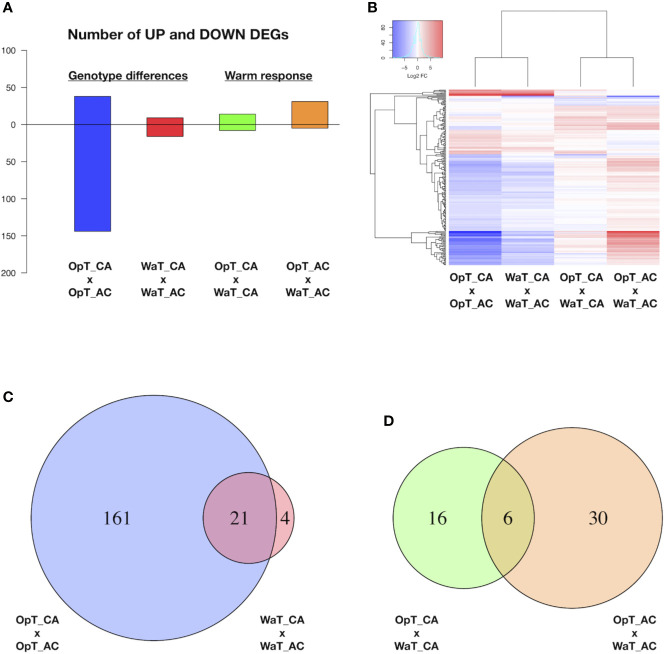
RNAseq analysis of two coffee genotypes under two temperature conditions. **(A)** Number of differentially expressed genes (DEGs) between coffee genotypes, cvs. Acauã and Catuaí, at stated temperature (OpT or WaT; named “genotype differences” in figure) and between the same cultivar at different temperatures (named “warm response”). **(B)** Heat map with up and down-regulated DEGs shown in A. **(C)** Venn diagram showing the DEGs found between coffee genotypes at OpT and WaT conditions (genotype differences) highlighting that the warm temperature causes a drastic reduction on DEGs. **(D)** Venn diagram showing the DEGs responsive to WaT for each coffee genotype (warm response). Details of DEGs, including genome identification, annotation and expression analysis, are available in **Table S2**. Labels: OpT, optimal temperature (23/19°C, day/night); WaT, warm temperature (30/26°C, day/night); CA, cv. Catuaí; AC, cv. Acauã. OpT_CA, WaT_CA, and WaT_AC were composed of five repetitions each, whereas OpT_AC had four repetitions.

The general response of plants to temperature stress involves multiple biological processes including transcriptional reprogramming and changes in cellular/physiological processes (Mittler et al., 2012; Barah et al., 2013). Since the present results indicate that coffee cv. Acauã and cv. Catuaí possess regulatory differences in leaves (**Figure 1**) in response to warming, we conducted a global transcriptome analyses to characterize the capacity of these coffee genotypes to respond to elevated temperatures through molecular regulatory pathways. We performed RNAseq analysis on leaf tissue from the two coffee cultivars and characterized DEGs within and between the two cultivars in response to warming (**Figure 2**).

Two types of RNAseq analyses were made. One analysis compared gene expression between the two coffee cultivars at a select temperature (OpT; WaT), which revealed DEGs related to transcriptional differences between genotypes at a given growth temperature (**Figure 2A**). The second analysis examined DEGs within a genotype in response to different temperature conditions, which revealed genotypic-dependent DEGs in response to WaT (**Figure 2A**). A heat map shows the expression levels of DEGs ranging between −10 to +10 fold changes in expression, and presents two visible patterns; most of the DEGs were down-regulated in cv. Acauã in relation to cv. Catuaí at a fixed growth temperature, whereas most of DEGs responsive to WaT were up-regulated in both genotypes (**Figure 2B**). The annotation and fold expression details of all DEGs are provided in **Table S2**. In total, 186 DEGs were found when comparing gene expression of cv. Acauã to cv. Catuaí (**Figure 2C** and **Table S2**); 161 DEGs were observed at OpT (130 down- and 31 up-regulated), 4 DEGs were detected exclusively at WaT (2 down- and 2 up-regulated), and 21 DEGs were shared across the two temperature regimes (14 down- and 7 up-regulated).

To functionally characterize the DEGs, we performed analysis of gene ontology (GO) and pathways using blast2GO (Götz et al., 2008), KEGG toolkits (Kanehisa et al., 2016), and AgriGO (Yan et al., 2017). The main categories found for DEGs were related to carbohydrate and protein metabolism (**Figure S1A**), while biological process and molecular function of DEGs were related to biotic stimulus, defense response, oxi-reduction, and oxidoreductase activity (**Figure S1B**). In agreement, the pathway differences of starch and sugar metabolism (**Figure S2**) showed DEGs related to enzymes including sucrose-6-phosphate (EC 3.2.1.26), UDP-glucose (EC 2.4.1.13), and trehalose (EC 3.1.3.12). Thus, coffee genotypes at 1-year of age presented differences in gene transcription at optimal growth temperatures that are related to energy metabolism. In contrast, the number of DEGs between the two genotypes were reduced drastically when cultivars were placed under WaT (**Figure 2C**) suggesting that many of the intraspecific transcriptional differences were restricted to OpT conditions.

Our analyses of coffee genotypes revealed a total of 52 DEGs in response to WaT (**Figure 2D** and **Table S2**), in which 16 DEGs occurred exclusively in cv. Catuaí (9 up- and 7 down-regulated) and 30 in cv. Acauã (26 up- and 4 down-regulated) while six DEGs were in common between the two genotypes (5 up- and 1 down-regulated). We performed gene annotation and GO analyses of DEGs (**Table S2**and **Figure S3**) which revealed that the most enriched GO category and biological process was related to carbohydrate metabolism (**Figure S3**). Indeed, three of these DEGs represent enzymes that are part of the carbohydrate pathway of starch and sucrose metabolism (**Figure S4**); GRANULE-BOUND STARCH SYNTHASE (EC 2.4.1.242/Cc08_g16970), GLUCOSE-1-PHOSPHATE ADENYLTRANSFERASE (EC 2.7.7.27/Cc02_17340), and ALPHA AMYLASE (EC 3.2.1.1/Cc06_g08480).

In cv. Acauã, we found additional molecular pathways represented by DEGs in response to warmer temperatures that included plant hormone signal transduction and carbon metabolism (**Figures S5** and **S6**), represented, respectively, by the DEGs *ABA RESPONSIVE ELEMENT BINDING FACTOR* (*ABF*; Cc10_g04070) and *PYRUVATE PHOSPHATE DIKINASE* (*PPDK*; Cc03_g02730; EC:2.7.9.1). These results are in agreement with the higher number of DEGs responsive to WaT found in cv. Acauã (**Figure 2D**).

To compare expression differences between genotypes in response to WaT and possible implications on metabolic regulatory pathways, we selected 10 DEGs to check gene expression (out of 52) related to energy metabolism and temperature responses, in which six were shared and three exclusive to cv. Acauã and one exclusive to cv. Catuaí (**Figure 2D**). Nine of these 10 DEGs showed upregulation in at least one coffee genotype in response to WaT, whereas the *SMALL HEAT SHOCK* (*sHSP-like*; Cc11_g16360) was only downregulated in both. *PCC13-62* was the only DEG that presented expression difference between genotypes at the WaT conditions. The RNAseq results for these DEGs were validated by RT-qPCR, which showed similar expression trends for the 10 DEGs (**Figure S7**, see **Table S3** for statistics).

**Figure 3:**
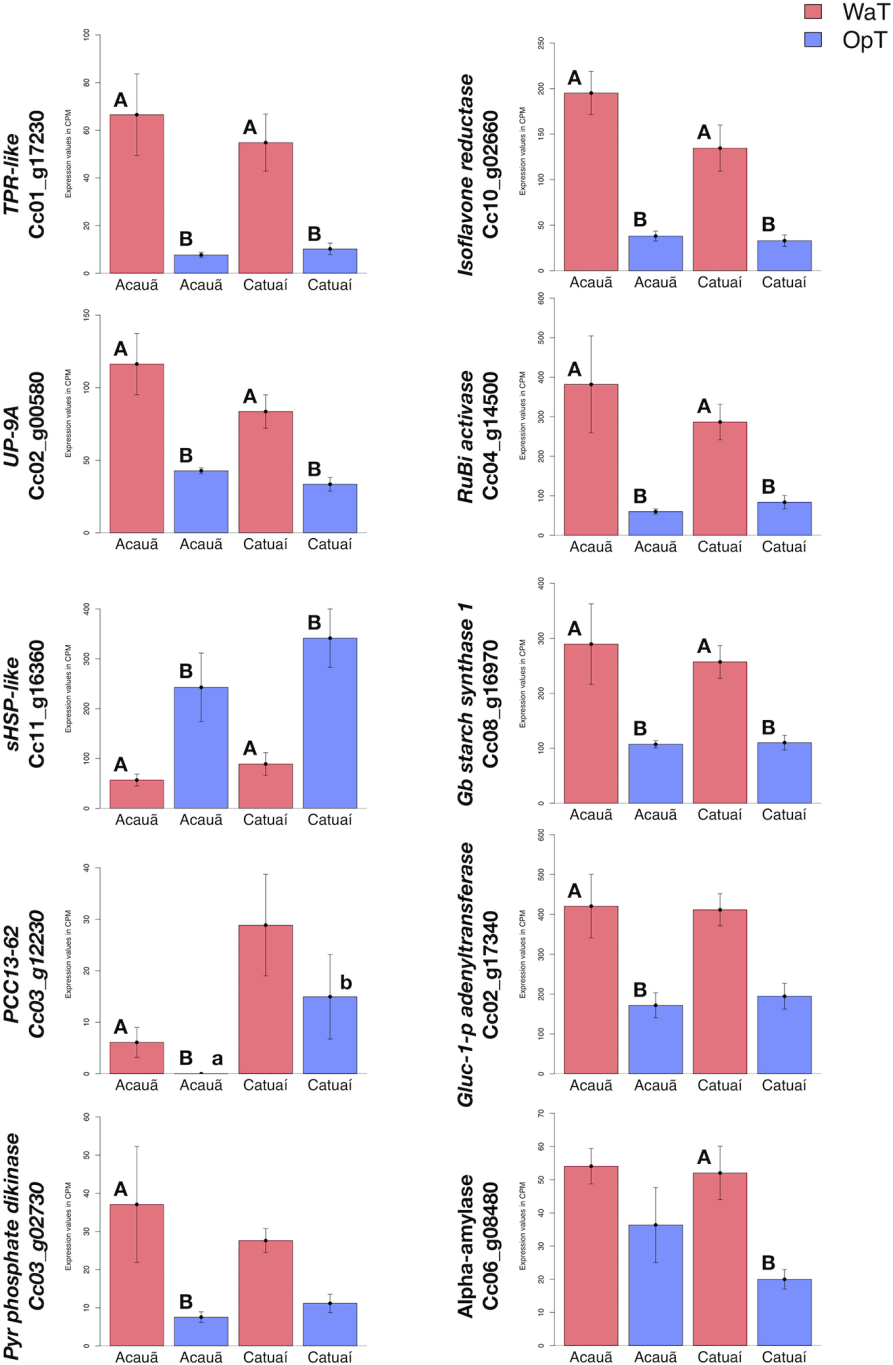
RNAseq expression analysis of warm-responsive DEGs related to energy metabolism and thermotolerance. Based on analysis of DEGs (**Figure 2D** and **Table S2**), we evaluated RNAseq expression of ten DEGs related to energy metabolism and thermotolerance; six DEGs that were shared between the two genotypes and four that were exclusive to one of the two genotypes, three in cv. Acauã and one in cv. Catuaí, respectively: the TPR-like (Cc01_g17230), isoflavone reductase (Cc10_g02660), UP-9A (Cc02_g00580), RuBisCO activase (RuBi activase; Cc04_g14500), small heat shock protein like (sHSP-like; Cc11_g16360), and granule-bound starch synthase 1 (Gb starch synthase 1; Cc08_g16970); desiccation-related_protein_PCC13-62 (PCC13-62; Cc03_g12230), glucose-1-phosphate adenyltransferase (Gluc_1_p_adenyltransferase; Cc02_17340); pyruvate phosphate dikinase (PPDK; Cc03_g02730); and alpha-amylase (Cc06_g08480). Statistical analyses were performed comparing the same coffee genotype at different temperatures (capital letters) and comparing different genotypes at the same temperature (small letters). Differences were considered significant at p<0.05 (see **Table S2** for details). Error bars represent standard errors. Labels: OpT (blue columns), optimal temperature (23/19°C, day/night); WaT (red columns), warm temperature (30/26°C, day/night). Catuaí both under OpT or WaT had five biological repetitions, as well as Acauã under WaT. Acauã under OpT had four biological repetitions. Error bars depict the standard error.

DEGs between coffee genotypes (**Figure 2D**) suggest the existence of a conserved mechanism in response to WaT and also exclusive pathways, both mainly related to carbohydrate metabolism control (**Figures S1** and **S3**). For example, *GRANULE-BOUND STARCH SYNTHASE 1* (Cc08_g16970) was up-regulated in both coffee genotypes in response to WaT (**Figures 3** and **S4**). However, other regulatory genes such as *GLUCOSE-1-PHOSPHATE ADENYLTRANSFERASE* (Cc02_17340) was only observed up-regulated in cv. Acauã whereas *ALPHA-AMYLASE* (Cc06_g08480) was only up-regulated in cv. Catuaí. These results demonstrated that the transcriptional pathways related to energy metabolism are affected by warmer temperatures in a genotype-dependent manner, consistent with results comparing different coffee species (Bertrand et al., 2015).

### Warmer Temperature Affects Sugar and Protein Content of Coffee Genotypes

**Figure 4:**
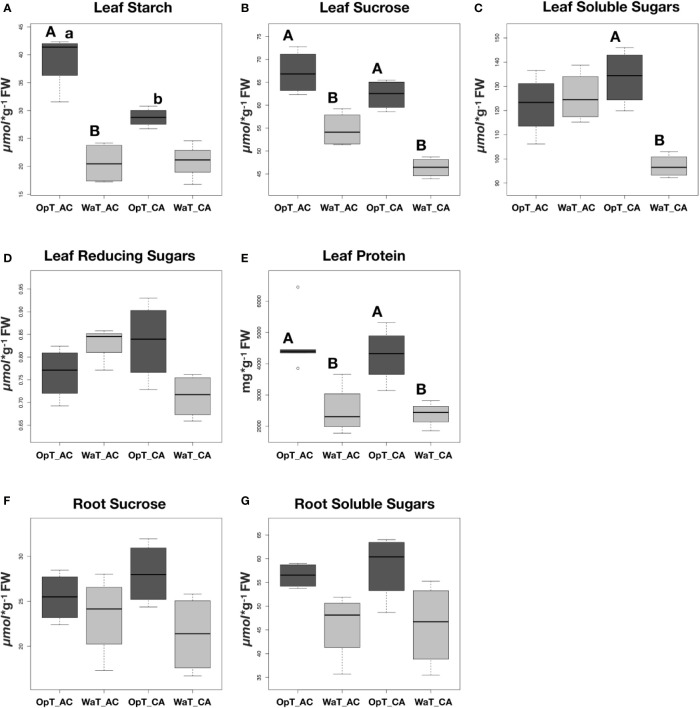
Boxplot analyses of different sugars and protein content in leaves and roots of two coffee genotypes under two temperature conditions. Coffee genotypes, cv. Acauã (AC) and Catuaí (CA) were subjected to two temperature conditions (OpT and WaT) and the starch content **(A)**, sucrose **(B)**, soluble sugars **(C)**, reducing sugars **(D)**, and protein **(E)** were determined in leaves. The content of sucrose **(F)** and soluble sugars **(G)** were also measured in roots. Statistical analyses were performed comparing the same coffee genotype at different temperatures (capital letters) and comparing different genotypes at the same temperature (small letters). Differences were considered significant at p<0.05 (see **Table S4** for details). Labels: OpT, optimal temperature (23/19°C, day/night); WaT, warm temperature (30/26°C, day/night); CA, cv. Catuaí; AC, cv. Acauã. Each box represents the distribution of values for eight plants in a given treatment.

Based on previous results from transcriptional analyses, we investigated whether warmer growth temperatures could affect the sugar content in a genotype-dependent manner (**Figure 4**). With several noted exceptions, i.e. soluble and reducing sugars in cv. Acauã leaves, both coffee genotypes showed similar patterns with higher carbohydrate and protein content in leaves at OpT compared to WaT growth conditions (**Figure 4**). Statistical analyses revealed specific differences in response to WaT (see **Table S4**), including a significant drop in leaf starch content in cv. Acauã at WaT (p<.0001; **Figure 4A**), whereas starch content decreases in cv. Catuaí was significant at a much lower probability level (P<.0628). Accordingly, cv. Acauã showed higher leaf starch levels at OpT conditions than cv. Catuaí, but leaf starch content dropped to a similar low level in both cultivars when exposed to WaT conditions.

For leaf sucrose content, both genotypes showed similar content at OpT conditions (**Figure 4B**), but the reduction in sucrose content was significantly greater for cv. Catuaí (P<.003) when compared to cv. Acauã (P<.018). Leaf soluble sugars were only reduced in cv. Catuaí leaves under WaT conditions (**Figure 4C**), whereas reducing sugar content was similar in both cultivars at both growth temperatures (**Figure 4D**). Leaf protein content of cv. Acauã and cv. Catuaí mirrored one another with a marked decrease in protein content under warm stress conditions (**Figure 4E**). By comparison, roots did not exhibit a difference in sucrose or soluble sugars content due to growth temperature or genotype identity (**Figures 4F, G**).

Thus, both the carbohydrate and protein content analyses revealed that moderate increases in growth temperature impacts coffee leaves at the metabolic level. Moreover, the specific differences between coffee genotypes in response to elevated temperatures, especially starch and soluble sugars, demonstrated that temperature effect is genotype-dependent.

## Discussion

The capacity of plants to regulate its internal temperature in a variable environment has important implications on the net carbon maintenance to an optimum photosynthesis performance (Wahid et al., 2007; Michaletz et al., 2015). Consequently, this thermoregulation is a very important issue for crop development and production (Janni et al., 2020). Coffee genotypes differ in their physiological and molecular responses to WaT stress reflecting an intraspecific genetic variability that once revealed would be useful for breeding (DaMatta, 2018; Damatta et al., 2018). To explore this variation and to better understand thermotolerance mechanisms, we compared the effect of WaT on the physiological, transcriptional, and metabolic status of two commercial coffee genotypes.

The present study showed striking differences in Tleaf between genotypes in response to an increased temperature, but similarities in stomatal conductance, leaf transpiration, and photosynthetic rate (**Figure 1**). This is a similar behavior observed for other species in part explained by differences at leaf traits (Michaletz et al., 2015; Michaletz et al., 2016), which could suggest the same for the compared coffee genotypes. However, we interpreted the physiological results as genotype intrinsic responses to temperature because: i) both coffee genotypes belong to the same species (*C. arabica*) with no clear visual morphological differences at first; ii) both genotypes were also compared to its own background, which showed different rates for each physiological parameter. This is in agreement with Bertrand et al. (2015), which compared different coffee species (*C. arabica*, *C. canephora* and *C. eugenioides*) and found no significant or few differences for growth rates at similar temperature conditions but with no comparisons of leaf traits. Moreover, our findings are in agreement with recent studies that describe photosynthetic stability, as well as thermoregulatory differences, between coffee genotypes in response to increased growth temperatures (Bertrand et al., 2015; Martins et al., 2016; DaMatta et al., 2019). Comparative studies identifying warm tolerant coffee genotypes are scarce and our results show that, based on the established experimental conditions, cv. Acauã appears to better regulate Tleaf than cv. Catuaí (**Figure 1** and **Table S1**). This contrasting result was used to explore the underlying molecular intraspecific pathways related to thermotolerance.

One noted advantage of RNAseq analysis is the global examination of all expressed genes under defined environmental/developmental conditions. This allows a detailed examination of the entire transcriptome to reveal stimulus-driven mechanisms (Lowe et al., 2017). In the present study, the higher number of DEGs found between coffee genotypes at OpT compared to WaT conditions was unanticipated (**Figure 2**) and demonstrates that both cultivars show a similar transcriptional response to warmer temperatures. Regarding this, it is tempting to suggest a bottleneck effect of transcription in response to WaTs. Yu et al. (2007) coined the term bottleneck to refer to highly centrality regulatory nodes that play key roles in mediating communication within a given network. Here, warmer temperatures would function as a centralizing point of transcription, possibly converging similar responses for thermoregulation and relocating energy resources between pathways, thereby acting as a constraint of variability. This is in accordance with the concept of hubs (or bottlenecks) in molecular signaling networks (Dietz et al., 2010), and the genotype-dependent effect of ambient temperature in plant plasticity (Ibañez et al., 2017; Zhu et al., 2018). Thus, stress-imposed constraints impact the energy costs for plant development limiting phenotypic plasticity (Auld et al., 2010; Murren et al., 2015) and challenging the selection of crop genotypes resilient to climate change (Pereira, 2016).

In response to WaT, we found six DEGs shared by the two examined coffee genotypes (**Figure 2D** and **Table S2**), whose expression trends were validated by RT-qPCR (**Figure S7**). These results indicate a core conservative thermoregulatory mechanism within the coffee genotypes. The homologs of *SMALL HEAT SHOCK PROTEINS* (Cc11_g16360) are triggered in response to stress and during the ripening process, acting as chaperones presenting a complex expression pattern (Ohama et al., 2016; Arce et al., 2018). RUBISCO ACTIVASE (Cc04_g14500), whose homologs enhance RUBISCO activity (Salvucci and Ogren, 1996), is up-regulated by WaT in coffee. RUBISCO is involved in carbon fixation during photosynthesis and it is negatively affected by increased growth temperature (Crafts-Brandner and Salvucci, 2000; Salvucci et al., 2001). Thus, the increased expression of *RUBISCO ACTIVASE* in coffee leaves was interpreted as a compensatory mechanism at WaT agreeing with photosynthetic rates that were unaffected by warmer temperature (**Figure 1C**). homologs of *ISOFLAVONE REDUCTASE* (Cc10_g02660) are involved in isoflavonoid synthesis, which are secondary metabolites related to lignin biosynthesis and pathogen defense (Shoji et al., 2002; Wang X. et al., 2006; Cheng et al., 2015). However, the direct relationship between isoflavone reductases and temperature stress has not been previously established. The DEGs *UP-9A* (Cc02_g00580) and *TPR-LIKE* (Cc01_g17230) appear to be a stress response related to sulfur deficiency. In *Arabidopsis*, homologs of *UP-9A* are putative interactors with ADP-glucose, which plays a key role in starch metabolism by converting glucose 1-phosphate to ADP-glucose (Crevillén et al., 2005). Homologs of *GRANULE-BOUND STARCH SYNTHASE* (Cc08_g16970) are involved in starch and sucrose metabolism pathways and in thermotolerance acquisition (Wang S. J. et al., 2006; Tian et al., 2018).

A series of DEGs responsive to WaTs were not shared between the two cultivars suggesting the possible existence of genotype-specific thermoregulatory mechanisms (**Figure 2D**). We observed that many of these DEGs are involved with carbohydrates and carbon regulatory pathways (**Figure S1** to **S6**). Within cv. Acauã, nearly twice as many DEGs were found in WaT versus OpT in comparison to WaT versus OpT DEGs in cv. Catuaí (**Figure 2D**). Of particular interest, cv. Acauã showed a downregulation of Cc10_g04070 in WaT, a gene specific to the pathway for stomatal closure *via* ABA regulation (**Figure S5**). As plants generally close stomata to prevent excess water loss in WaTs, a downregulation of a gene signaling the stomata to close may indicate a reduced sensitivity to slightly warmer temperatures.

The pathways that integrate temperature perception with physiological and metabolic regulation in plants largely depend on complex transcriptional networks (Ruan et al., 2010; Bita and Gerats, 2013). Our results showed a number of DEGs including *HSPs* and genes related to photosynthesis and carbohydrate metabolism, such as, *ATP synthases*, *starch synthases*, *amylases*, and others (**Table S2**), that are in line with published literature in other species. For example, upregulation of the *ATP synthase* subunit found in coffee is consistent with the findings for heat stress experiments in wheat, maize, and rice (Qin et al., 2008; Fernandes et al., 2008; Zhang X. W. et al., 2012). Moreover, other homologs were found from model plants such as *Arabidopsis* (Ohama et al., 2017) to C4 monocots grasses (Li et al., 2013) and also including the seagrass *Zostera marina* (Franssen et al., 2011) and red algae *Pyropia haitanensis* (Wang et al., 2018), which indicate an ancestral and conserved transcriptional response to heat stress. Moreover, also in agreement with our results (**Table S2**), heat transcriptome studies in crops showed a number of induced genes higher than the number of repressed ones (Qin et al., 2008; Fernandes et al., 2008; Zhang X. W. et al., 2012). However, the overall number of coffee DEGs was relatively lower, which could be explained by the different experimental conditions, mainly in the stress intensity and exposition time. In addition, *C. arabica* plants present specific characteristics such as the higher transcriptional and phenotypic homeostasis compared to others *Coffee* sp. (Bertrand et al., 2015) and great resilience of its photosynthetic-related processes that mitigate effects of warmer temperatures (Martins et al., 2016; DaMatta et al., 2019). Whereas, the relative low number of DEGs found comparing coffee cultivars (**Figure 2C**) can be explained by the reported narrow genetic basis of *C. arabica* (Scalabrin et al., 2020).

Interestingly, our findings show a correlation between a more robust transcriptomic response in cv. Acauã and a better control of its Tleaf and metabolic homeostasis when compared to cv. Catuaí. In opposite, Bertrand et al. (2015) comparing the allotetraploid *C. arabica* with its diploid parental, found higher transcriptional stability related to phenotypic homeostasis in response to temperature. This is reasonable because to optimize the described trade-off between leaf thermal stability and photosynthetic stability (Michaletz et al., 2016), plants must be able to perceive temperature changes and adjust expression and activity of related enzymes, i.e. RUBISCO (Crafts-Brandner and Salvucci, 2000; Salvucci et al., 2001). Alternatively, this transcriptional difference could be explained by the range of comparisons. On the global scale the Arabica transcriptome could be more homeostatic than its parental, but this is totally different if considered specific groups of genes such as those linked to *redox* activity (Bertrand et al., 2015). Similarly, comparing the transcriptional profiles between plants of the same species, but with different genotypes, we expect to find less differences and only the most evident differential intraspecific pathways will be highlighted, which was, in fact, observed (**Figure 2**).

From the physiological parameters photosynthetic rate and stomatal conductance in plants under OpT showed in **Figure 1** and the Tukey’s test result table (**Table S1**) we can see that, except for the last week (4), those parameters are not statistically different. We believe that those differences reported in the last week under OpT may be due to an uncontrolled long-term effect which elicits different metabolic responses as a genotype dependent effect. Interestingly, a co-variation of light and temperature was proposed for a better functioning of metabolism, which could affect the results obtained in growth chambers, more reproducible but different from a natural condition, where variable changing factors interact (Annunziata et al., 2018; Matsubara, 2018). In this way, our results showed fluctuations in physiological parameters under OpT conditions (**Figure 1**) difficult to be interpreted just considering the isolated temperature parameters. To support that, the differences found in photosynthetic rate and stomatal conductance in the last week of OpT was just a response to the lack of environment variability, we performed LMM regressions to access the linear relationship between photosynthetic rate and stomatal conductance (**Figure S8, box A**, **Table S5**) in different cultivars under the temperature treatments. We found that these parameters are significantly linear linked with each other during OpT treatment. However, Leaf Temperature is not correlated with photosynthesis nor conductance under OpT (**Figure S8, box B** and **C**, **Table S5**). The transpiration rate (**Figure S8, box D**, **Table S5**) is not statistically different between plants under OpT in any of the weeks (**Figure 1** and **Tables S1** and **S5**), although could be interpreted from **Figure 1**, otherwise. In this way, our results showed that fluctuations in physiological parameters under OpT (**Figure 1**) cannot be interpreted considering the temperature alone and is, probably, a long-term response to the chamber environment. This physiological variability was expected once we identified a higher number of DE genes under OpT than WaT (**Figure 2**).

Surprisingly, our results suggest that in coffee a mild increase in Tleaf does not cause an increase in stomatal conductance (**Figure S8 box C**). However, it shows that the increase in Tleaf decouples photosynthetic rate from stomatal conductance (**Figure S8 box A**) as reported by Urban et al. (2017) in *Pinus taeda* and *Populus deltoides*. This decoupling can promote the observed expression change in genes such *ALPHA AMYLASE* and *GB STARCH SYNTHASE* which, ultimately, will cause the reduction in leaf sucrose under WaT (**Figure 4B**). Additionally, this decoupling could be caused by the inability of RUBISCO ACTIVASE to increase RUBISCO carboxylation activity under WaT, even with its enhanced expression (**Figure 3**). In this scenario, the carbon assimilation would not be benefited from an increase in the stomatal conductance once RUBISCO would be constrained by its own activity rate, leading to the relative stability of photosynthetic rate across WaT (**Figure 1C**). Without any additional photosynthetic gain from an increase in stomatal conductance we would expect this lack of relationship between both physiological parameters. These physiological adjustments may be a strategy to optimize photosynthesis under environmental temperature gradient by the modulation of leaf biochemistry, (Wahid et al., 2007) mainly by the regulation of sugar metabolism.

Sugar metabolism is a complex and dynamic process strongly controlled by many pathways that once deregulated it usually affects the carbon and protein partitioning in plants (Rolland et al., 2006; Gutiérrez et al., 2007; Zhang M. Z. et al., 2012; Kölling et al., 2015). Because coffee genotypes differ in starch and sucrose metabolic pathways (**Figure S4**), we hypothesize that, in response to WaTs, different enzymes would be activated triggering changes in carbohydrate and protein content. For example, cv. Acauã could accumulate more starch in leaves than cv. Catuaí under OpT conditions, which would represent a carbohydrate reserve when unfavorable growth conditions are imposed. Our results demonstrated that there are differences in leaves regarding the sugar content, such as starch, sucrose and total soluble sugars (TSS), and in total protein content in response to warmer temperatures (**Figure 4**). These negative correlations between sugar content and temperature stress are in agreement with the report for *C. arabica* by Bertrand et al. (2015) and other species. For example, in maize (Boehlein et al., 2019) the increase of temperature decreased the abundance of mRNA related to biosynthesis of starch and storage proteins together with a faster rate of developmental program of endosperm. In sorghum (Jain et al., 2007), elevated growth temperature condition affects the temporal expression profiles of various genes involved in sugar cleavage and utilization, transport and starch biosynthesis leading to altered carbohydrate metabolism, starch deficiency and reduced microspores germination. Similar effects of temperature on the transcription profile related to energy metabolism and the sugar contents were reported for grapevine, which affected the berry ripening (Rienth et al., 2016), switchgrass (Li et al., 2013), and seaweed (Wang et al., 2018).

These considerations indicate that a similar core, interconnecting transcriptome and energy metabolism, is affected by elevated temperatures in plants, but such pathways evolved in different ways specific to each species to adjust the internal temperature to its living ambient (thermoregulation; Michaletz et al., 2015; Michaletz et al., 2016). Altogether, these comparisons reinforce our data that warmer temperature has a genetic and physiological impact on coffee leaves in a genotype-dependent manner and, once we revealed intraspecific differences, they might be used to understand aspects of thermotolerance and for breeding programs.

## Concluding Remarks

Coffee is a worldwide commodity, produced in over 80 countries and foundational to the economy of many regions through employment and trade (DaMatta and Ramalho, 2006). Climatic changes will not only affect the production of this high-value commodity, it will also elicit major economic and social repercussions. In this work, we showed an overall reduction in the number of DEGs between coffee genotypes under warmer temperature in comparison to OpT. Gene expression at OpTs was more diverse suggesting that these genotypes have variable baseline transcription. Moreover, we found specific genes responsive to warmer temperatures that could be involved in the temperature perception and response. These genes can be used as biomarkers for more accurate screenings of thermotolerant coffee genotypes. Some of these genes were related to sugar metabolism pathways and, although our findings demonstrate potential differences in starch and sucrose metabolic pathways along with variable physiological responses among cultivars, more studies are required to substantiate patterns of coffee thermotolerance. Our results confirm the need to search and develop more climate-adaptive coffee varieties as existent *C. arabica* L. cultivars may not possess the genetic variability needed to ensure consistent and profitable production of coffee in warmer temperatures (Lashermes et al., 1999; Anthony et al., 2001; Cubry et al., 2008; Scalabrin et al., 2020). Further investigation on intra and interspecific elevated temperature tolerance is warranted which may be critical to inform future introgression efforts of existing stress tolerance traits from other *Coffea* species into preferred Arabica genotypes.

## Data Availability Statement

The datasets presented in this study can be found in online repositories. The names of the repository/repositories and accession number(s) can be found in the article/**Supplementary Material**.

## Author Contributions

AC-J, BB, and PK conceptualized the project. RO, TR, CHC, and CFC designed and carried out the physiological analyses. BB prepared the RNAseq libraries that were analyzed by TR. TR and RO performed the bioinformatic analyses. LF and PK performed RT-qPCR analyses. RO, TR, and CHC designed and carried out the sugar and protein analyses. TR, VM, and RO performed the statistical analyses. RO and TR wrote the manuscript. LF, PK, and AC-J revised the manuscript.

## Funding

RO and AC-J were financially supported by the National Council of Scientific and Technological Development (CNPq) with the grants PDJ fellowship #167716/2017-4 and research fellowship # 311323/2016-2, respectively. The LFMP lab was partially supported by the National Institutes of Science and Technology of Coffee (INCT/Café) and CNPq.

## Conflict of Interest

The authors declare that the research was conducted in the absence of any commercial or financial relationships that could be construed as a potential conflict of interest.
