# Supplementary material for: Elevated Temperatures Impose Transcriptional Constraints and Elicit Intraspecific Differences Between Coffee Genotypes
Source: Front Plant Sci. 2020 Jul 21;11:1113. doi: 10.3389/fpls.2020.01113 (PMC7396624; doi:10.3389/fpls.2020.01113)
Supplement: Supplementary file 1 [file DataSheet_1.docx]

**Supplementary Material**


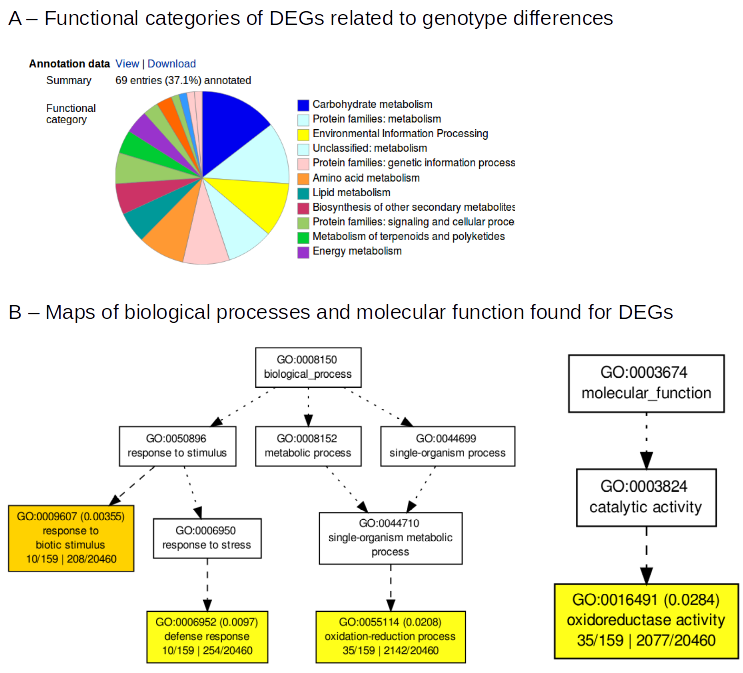


**Figure S1 – Functional categorization of the DEGs found between coffee genotypes at stated temperature**. A) The main Gene Ontology (GO) categories found for DEGs related to “Genotype differences” (Fig. 2A and 2C) were determined using the BlastKOALA/KEGG tool (Kanehisa et al., 2016). B) The enriched GO terms and maps of biological process and molecular function related to the same DEGs were found using blast2GO on *Coffea canephora* gene models (Götz *et al.*, 2008) and AgriGO toolkit (Tian et al., 2017).


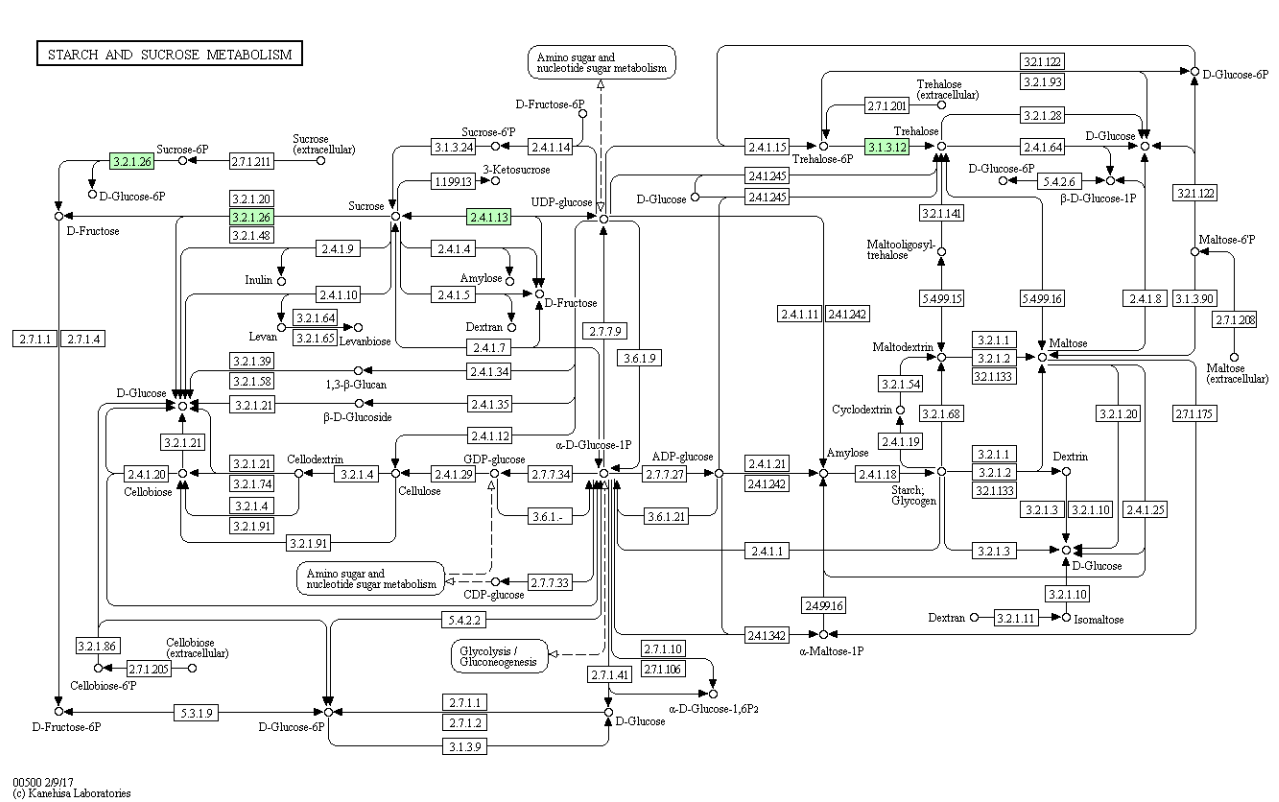


**Figure S2 – Three DEGs related to genotype differences at a stated temperature are involved in the pathway of starch and sucrose metabolism.** The figure was generated using the BlastKOALA/KEGG tool (Kanehisa et al., 2016) and revealed that between the DEGs related to genotype differences at a stated temperature (Fig. 2A and 2C) there are three relative to putative enzymes involved in the starch and sugar metabolism pathways (green in figure): Sucrose-6-phosphate (EC 3.2.1.26), UDP-Glucose (EC 2.4.1.13) and Trehalose (EC 3.1.3.12).


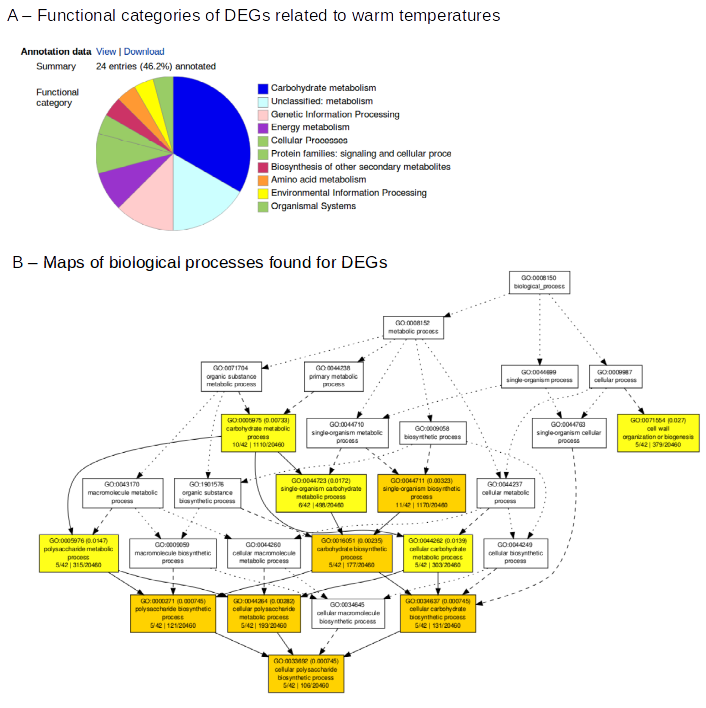


**Figure S3 – Functional categorization of the DEGs responsive to warm temperatures**. A) The main Gene Ontology (GO) categories found for DEGs related to warm temperatures (Fig. 2A and 2D) were determined using the BlastKOALA/KEGG tool (Kanehisa et al., 2016). B) The enriched GO terms and maps of biological process related to the same DEGs were found using blast2GO on *Coffea canephora* gene models (Götz *et al.*, 2008) and AgriGO toolkit (Tian et al., 2017).


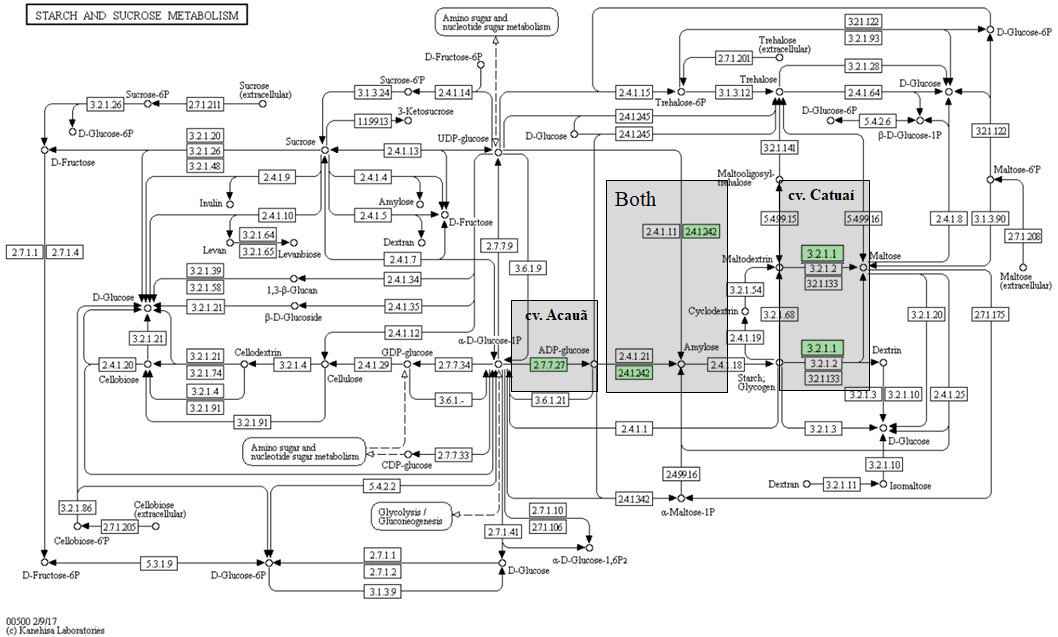


**Figure S4 – Three DEGs related to warm response in coffee cultivars are involved in the pathway of starch and sucrose metabolism.** The figure was generated using the BlastKOALA/KEGG tool (Kanehisa et al., 2016) and revealed that between the DEGs related to warm response in coffee genotypes (Fig. 2A and 2D) there are three relative to putative enzymes involved in the starch and sugar metabolism pathways (green in figure). The putative enzyme Granule-bound starch synthase (EC 2.4.1.242/Cc08_g16970) is differentially expressed for both coffee genotypes in response to warm temperatures, but Glucose-1-phosphate adenyltransferase (EC 2.7.7.27/Cc02_17340) is only up-regulated in cv. Acauã, whereas α-amylase (EC 3.2.1.1/Cc06_g08480) is only up-regulated in cv. Catuaí. These results suggest that transcriptional pathways related to energy metabolism are affected by warm temperatures in a genotype-dependent manner.


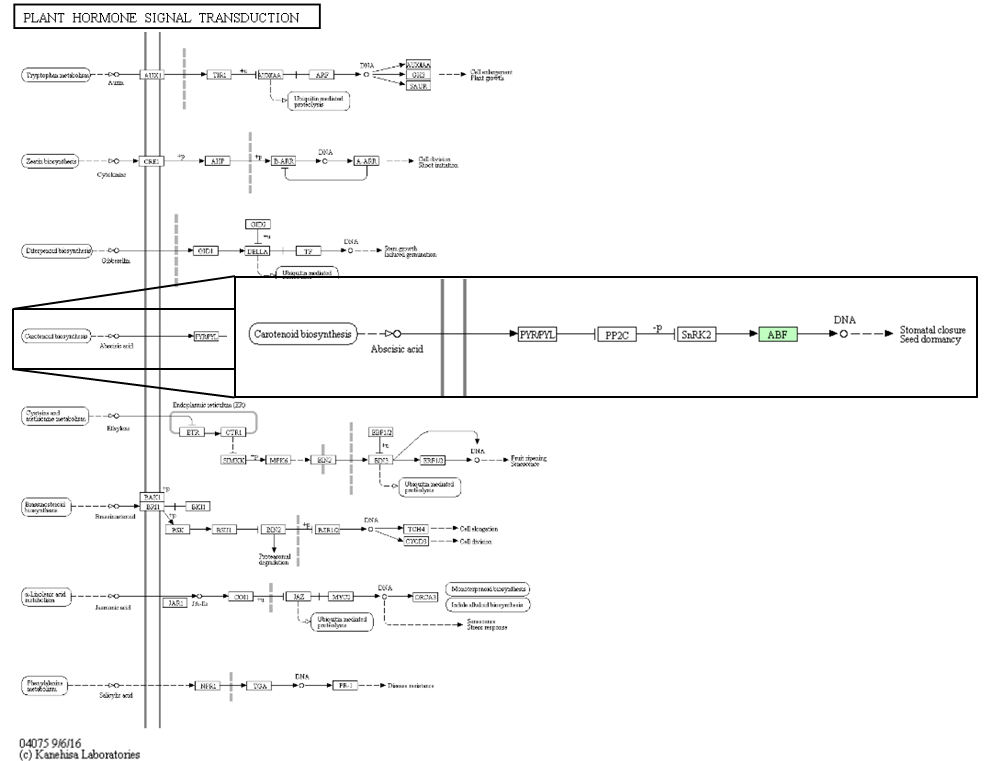


**Figure S5 – Analysis of the DEGs responsive to warm temperatures in cv. Acauã reveals differences in the plant hormone signal transduction pathway**. The figure was generated using the BlastKOALA/KEGG tool (Kanehisa et al., 2016) and shows down-regulation of the putative ABA responsive element Binding Factor (ABF; Cc10_g04070; in green) responsive to warm temperature and likely related to stomatal closure control.


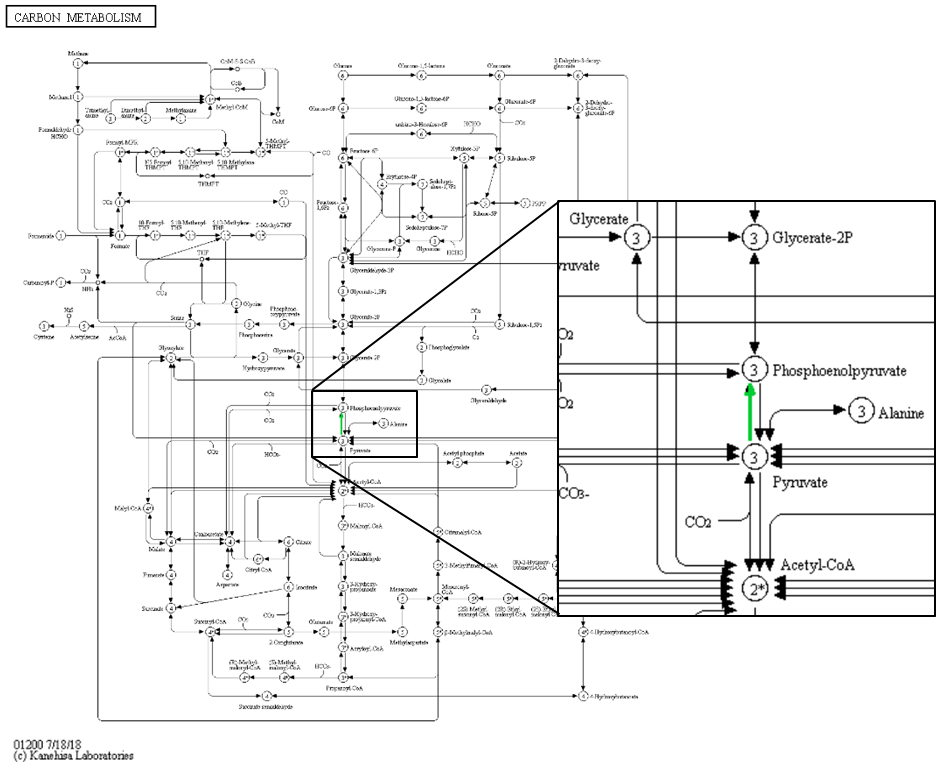


**Figure S6 – Analysis of the DEGs responsive to warm temperatures in cv. Acauã reveals differences in the carbon metabolism pathway**. The figure was generated using the BlastKOALA/KEGG tool (Kanehisa et al., 2016) and shows up-regulation of Pyruvate Phosphate DiKinase (PPDK; Cc03_g02730; EC:2.7.9.1; green arrow) in response to warm temperatures. This enzyme catalyzes the reaction of pyruvate to phosphoenolpyruvate during gluconeogenesis.


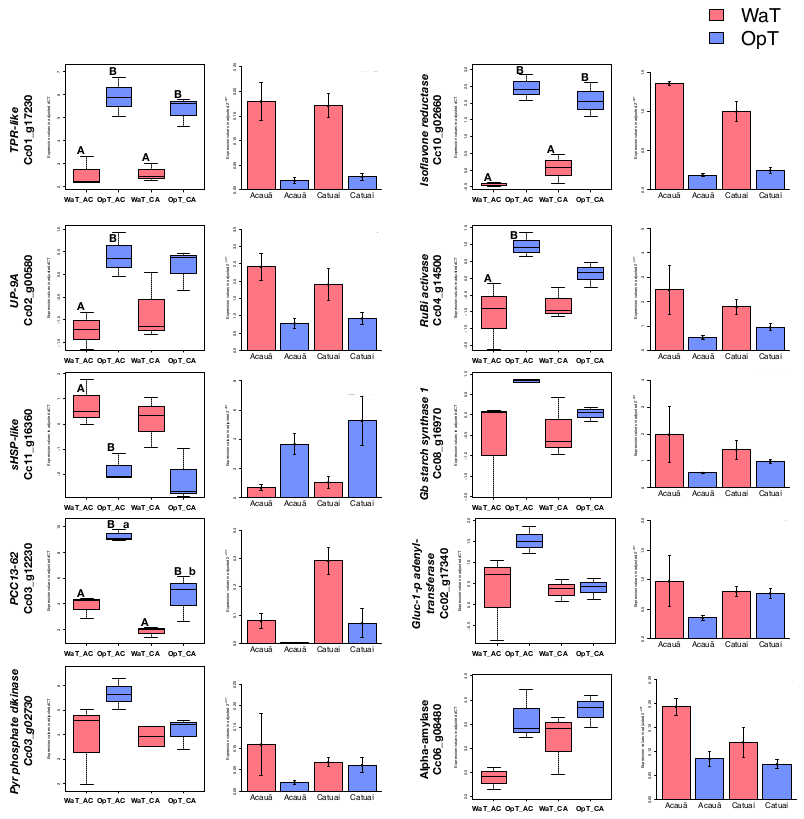


**Figure S7 – RT-qPCR expression analysis of ten selected DEGs identified by RNAseq.** The RT-qPCR was used to validate expression analysis of ten DEGs responsive to warm temperature identified by RNAseq (Fig. 2D). Expression values are shown as adjusted dCT (boxes, left side) and adjusted 2^⁻dCT^ method (right side). Ten DEGs related to energy metabolism and thermotolerance were selected, six shared between coffee genotypes, three exclusive to cv. Acauã and one to cv. Catuaí, respectively: the *TPR-like* (Cc01_g17230), *Isoflavone reductase* (Cc10_g02660), *UP-9A* (Cc02_g00580), *RuBisCO activase* (*RuBi activase;* Cc04_g14500), *Small Heat Shock Protein like* (*sHSP-like;* Cc11_g16360), and *Granule-bound starch synthase 1* (*Gb starch synthase 1;* Cc08_g16970); *Desiccation-related_protein_PCC13-62* (*PCC13-62*; Cc03_g12230), *Glucose-1-phosphate adenyltransferase* (*Gluc_1_p_adenyltransferase*; Cc02_17340) and *Pyruvate phosphate dikinase* (*Pyr phosphate dikinase;* *PPDK*; Cc03_g02730); *Alpha-amylase* (Cc06_g08480). Statistical analyses were performed comparing the same coffee genotype at different temperatures (capital letters) and comparing different genotypes at the same temperature (small letters). Differences were considered significant at p<0.05 (see Table S3 for details). Labels: OpT (blue columns) - optimal temperature (23/19°C, day/night); WaT (red columns) - warm temperature (30/26°C, day/night). AC - cv. Acauã; CA - cv. Catuaí IAC144. Each treatment was composed by 3 biological repetitions with two technical replicates each. Error bars depict the standard error.


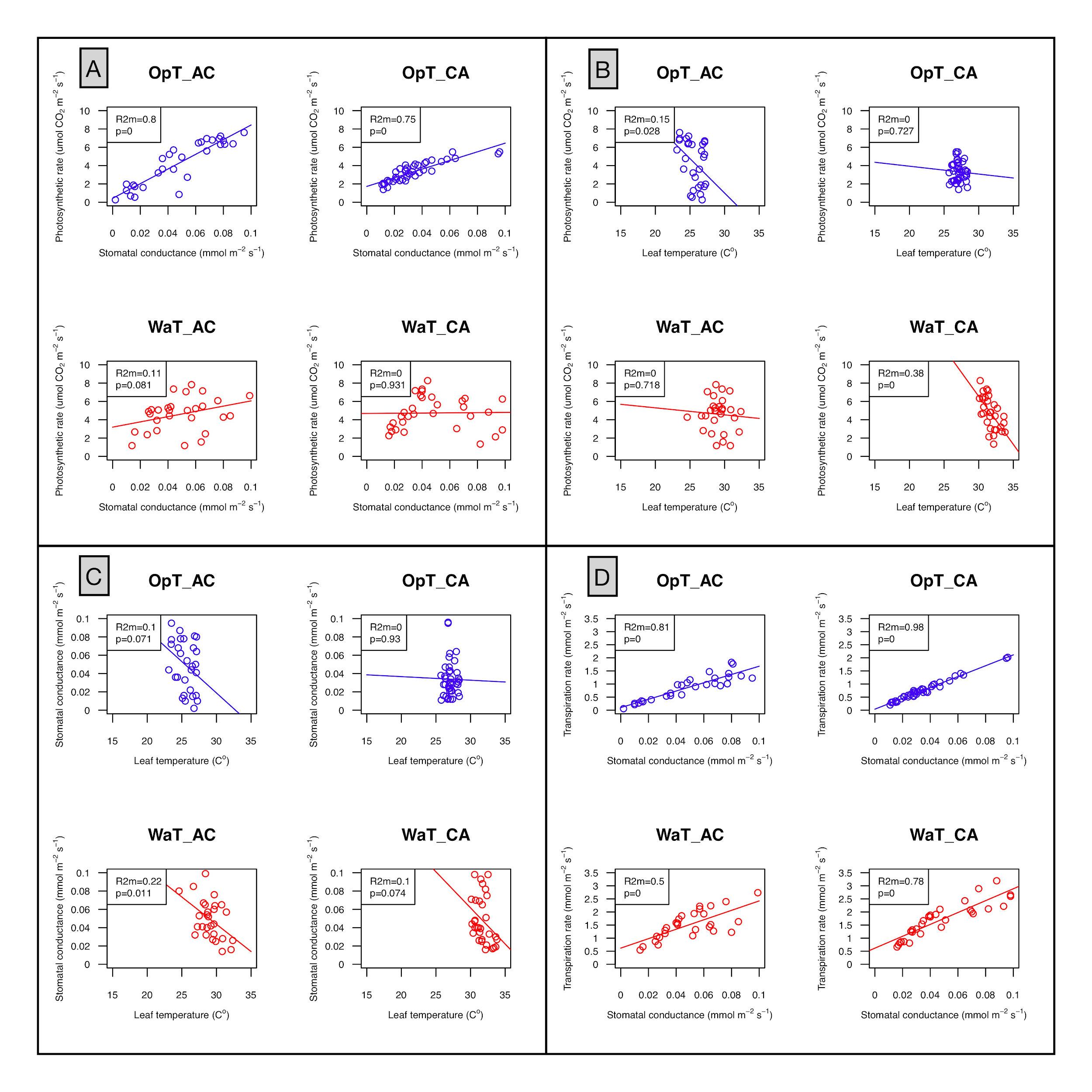


**Figure S8 - Pairwise relationships between physiological parameters estimated by Linear Mixed Effects Models.** Box A) Relationship between Photosynthetic rate and Stomatal Conductance. Box B) Relationship between Photosynthetic rate and Leaf Temperature. Box C) Relationship between Stomatal Conductance and Leaf Temperature. Box D) Relationship between Transpiration Rate and Stomatal Conductance. Blue represents optimal temperature while red represents Warm temperatures. R2m is the marginal R² of each model and p is the slope significance. OpT - optimal temperature (23/19°C, day/night); WaT - warm temperature (30/26°C, day/night); CA - cv. Catuaí IAC 144; AC - cv. Acauã. Each plot represents 40 readings nested within 10 plants.
